# Numerical and Physical Modeling of Liquid Steel Asymmetric Behavior during Non-Isothermal Conditions in a Two-Strand Slab Tundish—“Butterfly Effect”

**DOI:** 10.3390/ma16216920

**Published:** 2023-10-28

**Authors:** Adam Cwudziński, Jacek Pieprzyca, Tomasz Merder

**Affiliations:** 1 Department of Metallurgy and Metals Technology, Faculty of Production Engineering and Materials Technology, Czestochowa University of Technology, ArmiiKrajowej 19, 42-201 Czestochowa, Poland; 2 Department of Metallurgy and Recycling, Faculty of Materials Engineering, Silesian University of Technology, Krasinskiego 8 Str., 40-019 Katowice, Poland; jacek.pieprzyca@polsl.pl (J.P.); tomasz.merder@polsl.pl (T.M.)

**Keywords:** tundish, hydrodynamics, numerical simulations, water modeling, ladle shroud

## Abstract

This paper presents the results of studies on the occurrence of transient disturbances in the hydrodynamic system of a tundish feeding area and their effect on the casting process. In addition, the effect of changes in the level of superheating of the molten steel fed to the tundish on the evolution of the hydrodynamic system was analyzed. The studies were conducted with the use of a physical model of the tundish and a numerical model, representing the industrial conditions of the process of the continuous casting of steel. When a tundish is fed through a modified ladle shroud that slows down the momentum of the stream, this creates favorable conditions for the emergence of asymmetrical flow within the working tundish volume. The higher the degree of molten steel reheating in the ladle furnace, the stronger the evolution of the hydrodynamic structures in the tundish during the casting process.

## 1. Introduction

The continuous casting of steel is an important stage in steel product manufacturing. Approximately 162 million tons of crude steel were produced in April 2023 worldwide. Over 98% of semi-finished steel products are manufactured through continuous casting. Therefore, continuous casting technology is optimized and developed in terms of cost efficiency and improvements in the quality of cast strands. In the process of the continuous casting of steel, in which kinetics plays a special part, the “butterfly effect” is significant, in which even minor deviations from the physico-chemical or thermo-hydrodynamic steady state cause the occurrence of certain phenomena in a multiphase system which affect the final condition and the concast strand [1]. Disturbances occurring in the continuous casting process may be the effect of random interactions of components of a multiphase system, particularly when the system evolves in turbulent conditions. One of the essential components of a continuous casting machine is a tundish, with which additional metallurgic treatments can be successfully realized, specifically refining, microalloying, and molten steel reheating [2,3,4,5,6,7,8,9,10,11,12,13,14,15]. Whereas the tundish is a flow reactor, a unique thermo-hydrodynamic system forms inside its working space, as a function of the tundish’s shape, capacity, number of outlets, and installation of flow control devices or non-standard equipment, e.g., a vacuum chamber, annular porous permeable brick with a swirl block, swirling chamber, or multi-port ladle shroud [16,17,18,19,20,21,22,23,24,25,26,27,28,29,30]. In addition, the hydrodynamic system in the tundish is modified by the temperature gradient generated by the cooling of molten steel during the casting process, the inflow of a hotter or colder batch of steel into the tundish from the ladle, or the steel-reheating zones in the tundish [31,32,33,34,35,36,37,38,39,40]. The impact of natural convection is particularly observable in tundishes with acapacity of over 50 tons, fitted with turbulence inhibitors or fed via an advanced ladle shroud, which slow down the momentum of the supply stream. The thermo-hydrodynamic system in the tundish is susceptible to transient disturbances, particularly whenit is a turbulent system composed of local micro-systems characterized by individual levels of deviation from the steady state. This paper presents the results of studies of the occurrence of transient disturbances in the hydrodynamic system of the tundish feeding area and their effect on the casting process. In addition, the effect of changesin the level of superheating of the molten steel fed to the tundish on the evolution of the hydrodynamic system was analyzed. The studies were conducted with the use of a physical model of the tundish and a numerical model, representing the industrial conditions of the process of the continuous casting of steel.

## 2. Tundish Description 

**Figure 1:**
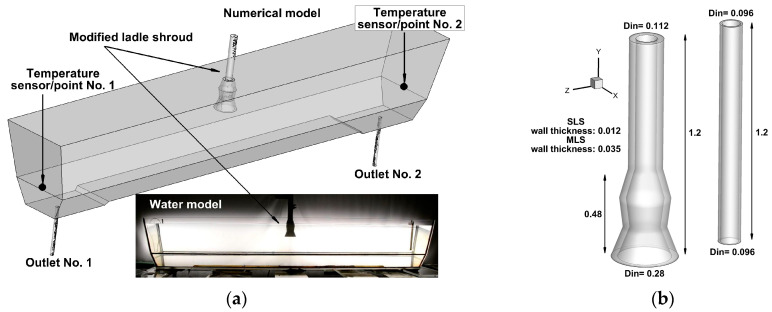
Two-strand tundish: (**a**) view of full-scale numerical model and 0.25-scale water model, (**b**) view of full-scale SLS and MLS (dimensions in m).

The tested object was a two-strand tundish designed for casting slabs (Figure 1a). The rated capacity of the tundish is 60 Mg. A detailed description and the dimensions of the tundish are presented in [41]. Within the feeding area, the tundish has a raised bottom that functions as a safety buffer to prevent the suction of casting powder to the continuous casting molds during the final stage of casting sequence. No flow control devices are installed in the tundish. The tundish is fed with molten steel entering through a ceramic ladle shroud. Two ladle shrouds were tested in the research project. The first shroud is a cylindrical standard ladle shroud (SLS) with aninside diameter of 0.096 m. The second is a dissipation shroud with the function of slowing down the tundish feed stream and the modification of liquid steel flow inside the tundish [42,43,44,45,46,47,48]. The shape and dimensions of the modified ladle shroud (MLS) are presented in this paper [49]. The length of both ladle shrouds is 1.2 m. A correction factor of 1.6 was added to the linear dimensions of the modified ladle shroud. Hence, the inside diameter of the shroud’s opening facing the ladle is 0.112 m, and it is 0.28 m on the side of the tundish (Figure 1b). There are two outlets in the bottom of the tundish. Figure 1a presents the layout of the sensors/measuring points at which a temperature variation in the continuous medium was registered during laboratory experiments and computer simulations. The points were located at 0.4 m above the bottom of the tundish, directly on the side walls.

## 3. Methodology

Computer simulations and laboratory experiments were carried out for the process of casting strand slabs with the dimensions of 1.5 m × 0.22 m, cast at a rate of 0.9 m/min. For a tundish fitted with a standard ladle shroud (SLS), the initial velocity is 1.368 m/s while kinetic energy of turbulence and dissipation rate of turbulence are 0.0187 m^2^/s^2^ and 0.0532 m^2^/s^3^, respectively. In the case of a modified ladle shroud (MLS), the initial velocity is 1.005 m/s and the kinetic energies of turbulence and dissipation are equal at 0.0101 m^2^/s^2^ and 0.0181 m^2^/s^3^, respectively. Kinetic energy and dissipation rate were calculated using Equations (1) and (2).

(1) $$k=0.01\cdot v_{in}{}^{2}$$

(2) $$\varepsilon =\frac{2k^{1.5}}{D_{in}}$$

where: *v_in_* is initial velocity of liquid steel (m/s), and *D_in_* is internal diameter of ladle shroud (m).

The initial molten steel temperature amounted to 1823 K (1550 °C). Table 1 presents all the research variants covered during this study. The computer simulations and laboratory experiments consisted of initiation of casting of a subsequent steel grade with a specified temperature exceeding that of the molten steel already inside the tundish. During the first simulations, the exchange of heat between the tundish and the environment was not taken into account. This was followed by a simulation of the casting process involving the transfer of heat from the molten steel to the environment. The final simulations not only included the exchange of heat between molten steel and the environment but also the drop of temperature of molten steel entering the tundish from the ladle, taking into account the natural cooling process of the cast steel. Type F residence time distribution (RTD) curve was registered during the study, which represents the mixing of two grades of steel with different chemical compositions that are cast in sequence one after the other. Based on the marker residence time distribution curves obtained for the working volume of the tundish, the time of intermixed steel between two grades of steel of different chemical compositions was determined.

### 3.1. Physical Modeling

Laboratory experiments were conducted on a model made of acrylic glass with a linear scale of 0.25. The rated capacity of the tundish model was 130 L. The model was fully automated so as to ensure repeatability and reach the required water flow rates for the casting rate considered in this paper, in accordance with Froude’s criterion.

(3) $${\left(\frac{u^{2}}{gL}\right)}_{m}={\left(\frac{u^{2}}{gL}\right)}_{p}$$

where: *L* is characteristic length (m), *u* is velocity of continuous medium (m/s), and *g* is acceleration of gravity (m/s^2^). 

Water was flowing into the model at the rate of 20.1 LPM. For the temperature gradients under consideration, according to Equation (4) [35], water temperature values were calculated, amounting to 5, 10, and 15 K for the values of 12, 23, and 35 K applied in the computer simulations, respectively.

(4) $$\Delta T_{water}=\frac{\beta_{steel}}{\beta_{water}}\cdot \Delta T_{steel}$$

where: *β_steel_* is coefficient thermal expansion of steel (0.000127 1/K), and *β_water_* is coefficient of thermal expansion of water (0.000295 1/K).

A solution of NaCl and KMnO_4_ was added to the ladle model during the experiments to record the changing concentration of the marker and visualize the mixing process during casting. The water salinity and temperature values were converted into non-dimensional values as per Equations (5) and (6) [50,51,52]. To standardize the results, real time was also converted to non-dimensional values (Dt), accounting for theoretical mean time of 394 s [52].

(5) $$T=\frac{T_{t}-T_{0}}{T_{inlet}-T_{0}}$$

(6) $$F=\frac{C_{t}-C_{0}}{C_{f}-C_{0}}$$

where: *T_t_* is temporary temperature, *T*_0_ is initial temperature inside tundish, *T_inlet_* is temperature of incoming fluid from ladle, *C_t_* is temporary tracer concentration, *C*_0_ is initial tracer concentration, and *C_f_* is final tracer concentration.

### 3.2. Mathematical Model

Numerical simulations were carried out using Ansys-Fluent software (version 12) for a full-scale tundish model. The numeric grid was made of approx. 1.5 million tetrahedral elements. Turbulence was presented through the realizable k-epsilon model. The single-phase numerical model was discussed in detail in [53]. The continuous medium in numerical simulations was molten steel with the followin gphysico-chemical properties: the viscosity is 0.007 kg/m·s, heat capacity of steel is 750 J/kg·K, and thermal conductivity of steel is 41 W/m·K. The numerical model accounted for the change in molten steel density as a function of temperature (Equation (7)).

(7) $$\rho =8300-0.7105T_{t}$$

The numerical simulations were conducted in two stages. First, the simulations were carried out for steady conditions, with or without heat exchange with the environment, and with the initial temperature of molten steel at 1823 K. Heat transfer from molten steel to the environment was evident from the heat fluxes on the walls and bottom of the tundish (−2600 W/m^2^) and the heat transfer from the free surface of molten steel was evident from the heat fluxes on the surface, covered with casting powder (−15,000 W/m^2^). Then, simulations were initiated for non-steady conditions in which a new grade of molten steel would be fed into the tundish at a temperature increased by the temperature delta values considered in the project, including or disregarding the temperature drop of molten steel in the ladle. The temperature drop (10 K and 20 K) in the ladle was described with a linear temperature profile and adapted to tundish’s inlet boundary condition. Theoretical mean time for the numerical model of the tundish was 874 s. The intermixing criterion can be characterized by different ranges, therefore it was assumed for research purposes that the transition length between the two grades of steel ranges from 0.2 to 0.8 of non-dimensional concentration [54,55]. During simulations, species model was adopted to solve the problem of mixing new grade steel with old grade steel. Both grades have the same physico-chemical properties. The equations forming the numerical model of steel flow weresolved by the pressure-based method via upwind discretization of the second order using the sequential solver. The Semi-Implicit Method for Pressure-Linked Equations-Consistent (SIMPLEC) algorithm was used for the description of the coupling of the pressure and velocity fields in the model being solved. The controlled level of residues was at least 10^−3^.

## 4. Results—Intermix Range Evolution 

### 4.1. Model Validation—Adiabatic Conditions

**Figure 2:**
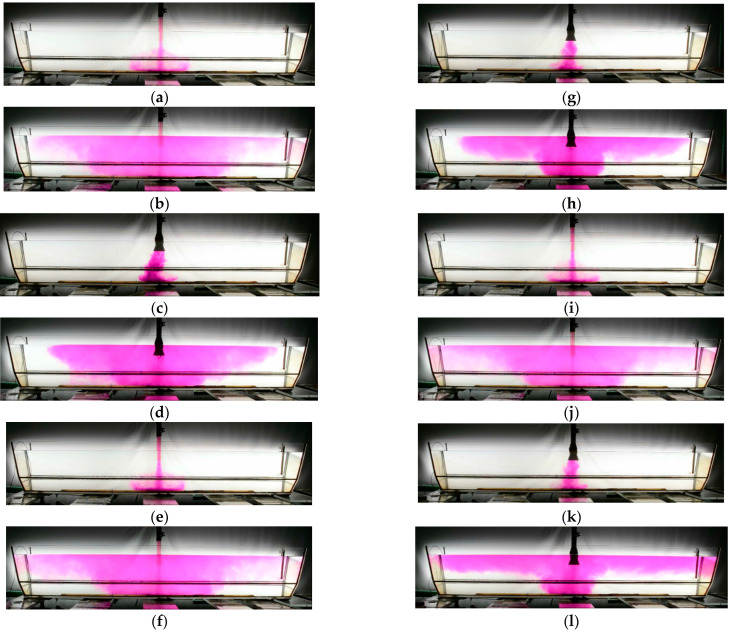
Water behavior inside tundish: (**a**) ΔT = 5 K, SLS, 0.012 Dt after the ladle gate opens, (**b**) ΔT = 5 K, SLS, 0.152 Dt after the ladle gate opens, (**c**) ΔT = 5 K, MLS, 0.012 Dt after the ladle gate opens, (**d**) ΔT = 5 K, MLS, 0.152 Dt after the ladle gate opens, (**e**) ΔT = 10 K, SLS, 0.012 Dt after the ladle gate opens, (**f**) ΔT = 10 K, SLS, 0.152 Dt after the ladle gate opens, (**g**) ΔT = 10 K, MLS, 0.012 Dt after the ladle gate opens, (**h**) ΔT = 10 K, MLS, 0.152 Dt after the ladle gate opens, (**i**) ΔT = 15 K, SLS, 0.012 Dt after the ladle gate opens, (**j**) ΔT = 15 K, SLS, 0.152 Dt after the ladle gate opens, (**k**) ΔT = 15 K, MLS, 0.012 Dt after the ladle gate opens, and (**l**) ΔT = 15 K, MLS, 0.152 Dt after the ladle gate opens.

Figure 2 presents the behavior of the continuous medium in the acrylic glass model of the tundish from the initiation of the ladle opening. Water dyed with KMnO_4_ imitates a new grade of molten steel. For the temperature gradients under consideration, the same characteristic casting times were chosen to represent the hydrodynamic structure forming in the tundish. Irrespective of the temperature gradient in the first phase of pouring water into the tundish with a standard ladle shroud, the water stream moves symmetrically against the shroud, running towards the outlets after touching the bottom of the tundish (Figure 2a,e,i). After 1 min, the effect of temperature is more prominent (Figure 2b,f,j). With a temperature difference of 15 K, the surface area of the dyed liquid at the bottom of the tundish decreases, which is an indication of the horizontal stratification of recirculating streams in the tundish model (Figure 2j). In a tundish with a modified ladle shroud, the slowdown of the feeding stream is primarily observed, as it covers a smaller distance from the shroud axis after 5 s (Figure 2c,g,k). Strong non-steadiness can also be observed in the behavior of the feeding stream, which is typical of this type of flow control device. The feed stream tends to behave asymmetrically, with a tendency to prefer movement towards outlet No. 1. It can be noted here that for a temperature delta of 10 K, the stream is moving quite symmetrically compared to the shroud axis (Figure 2g,h). The more asymmetry there is at the point of the feed stream touching the bottom of the tundish, the more asymmetrical the structure will develop in the working volume of the tundish (Figure 2d). In the case of a shroud that slows down the momentum of the feed stream, the impact of the temperature gradient is particularly prominent (Figure 2h,l). For the delta value of 15 K, the horizontal stratification of recirculating streams is clearly visible, occurring as a consequence of the difference in density (Figure 2l). The hotter batch of water flows on top of the colder water residing in the tundish. 

**Figure 3:**
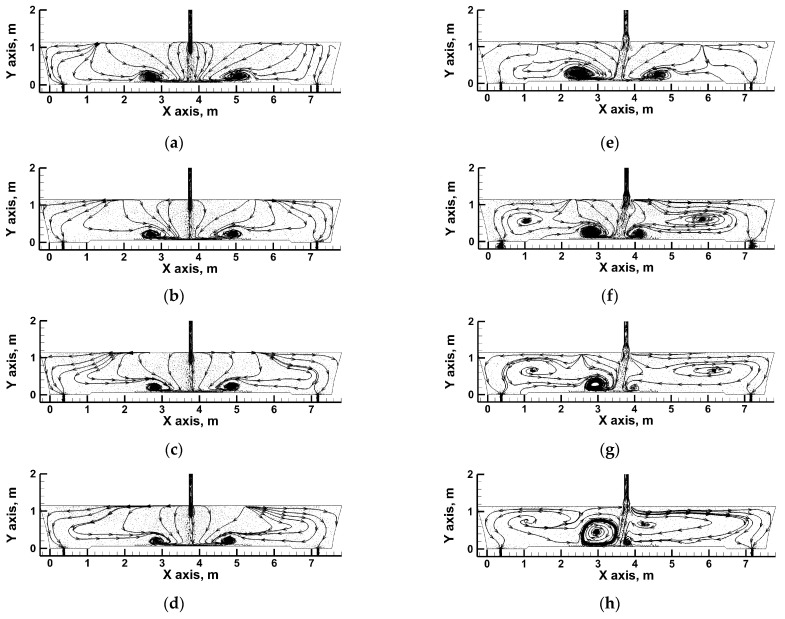
Liquid steel behavior inside tundish: (**a**) SLS, steady-state conditions; (**b**) SLS, transient conditions: 0.55 Dt and ΔT = 12 K; (**c**) SLS, transient conditions: 0.55 Dt and ΔT = 23 K; (**d**) SLS, transient conditions: 0.55 Dt and ΔT = 35 K; (**e**) MLS, steady-state conditions; (**f**) MLS, transient conditions: 0.55 Dt and ΔT = 12 K; (**g**) MLS, transient conditions: 0.55 Dt and ΔT = 23K; (**h**) MLS, transient conditions: 0.55 Dt and ΔT = 35 K.

Figure 3 presents the hydrodynamic system on the plane crossing the tundish’s feeding and emptying areas. In tundishes both fitted with a standard ladle shroud and with a modified ladle shroud, characteristic molten steel recirculation areas are present at the bottom, approx. 1 m away from the feed stream axis (Figure 3a,e). The steel recirculation area is formed through the interaction of backflows with the streams feeding the tundish. In the simulations for steady conditions without the exchange of heat with the environment and with a uniform temperature field, the main factors defining the hydrodynamic system are as follows: the momentum of the feed flux; and the tundish workspace geometry. Within the area of our analysis, the steel streams fall towards the bottom and the outlets fall mostly vertically (Figure 3a,e). However, when the casting of a subsequent grade of steel with a higher temperature is initiated, a temperature gradient occurs, along with the horizontal stratification of the circulation streams approx. 2 m or less from both of the tundish’s outlets (Figure 3b). This effect is the more prominent the higher the temperature difference between the molten steel in the tundish and the steel batch flowing out of the ladle (Figure 3c,d). In the case of a tundish with a modified ladle shroud, a tendency was discovered in the simulation towards steady conditions for the feed stream to turn toward outlet No. 1, consistent with the image recorded during the laboratory experiments. With the uniform temperature field, despite the asymmetry in the behavior of the feed stream, the hydrodynamic structure formed in the tundish is almost identical on both sides of the feed stream axis. If the asymmetry in the distribution of the feed stream persists, the emerging temperature gradient will not only intensively modify the hydrodynamic structure through the horizontal stratification of the circulation streams approx. 2.5 m or less away from the outlet nozzles towards the feeding area (Figure 3f–h). The initiation of a casting process for a batch of steel with a higher temperature in a system with a tendency for the feed flux to be asymmetrical strongly enhances the “butterfly effect”, presenting itself as the evolution of and increase in the steel recirculation area at the bottom of outlet No. 1’s side of the tundish. With the temperature difference equaling 35 K, the recirculation area reaches almost 2/3 of the metal column height, while at the same time, the circulation of molten steel at the bottom of outlet No. 2’s side fades almost completely after 480 s (Figure 3h). 

**Figure 4:**
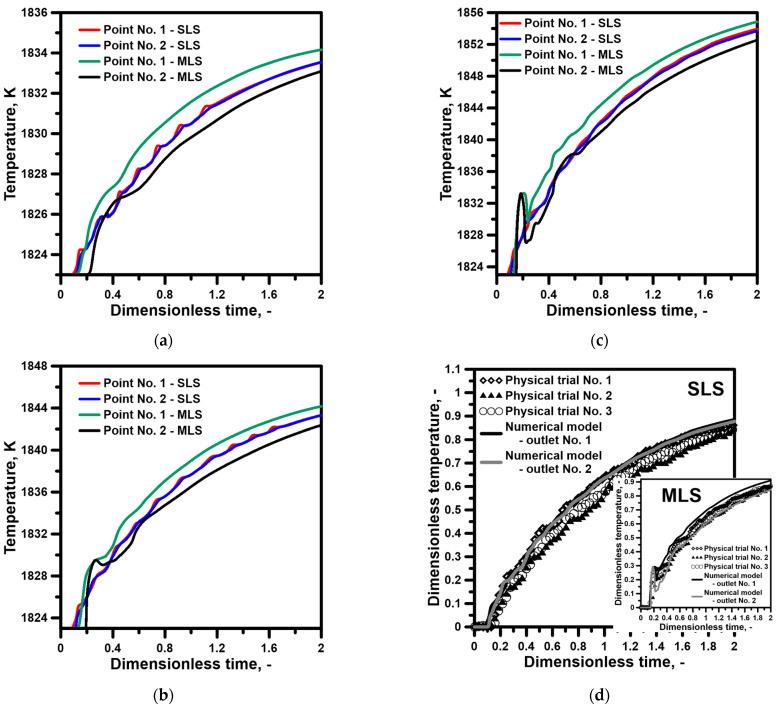
Distributions of temperature inside tundish for both ladle shrouds (numerical simulations): (**a**) liquid steel ΔT = 12 K, (**b**) liquid steel ΔT = 23 K, (**c**) liquid steel ΔT = 35 K, (**d**) model validation.

The images obtained during the laboratory experiments and computer simulations, characterizing the hydrodynamic structure, are the basis for the qualitative assessment of the tendencies thatmay be expected during a continuous casting process in the tundish depending on the type of ladle shroud used and the degree of the superheating of the molten steel. The first stage of the study also comprised the mutual validation of the applied research tools, the numerical model, and the water model. Figure 4 presents the distribution of temperature at measuring points 1 and 2. For a tundish with a standard ladle shroud, the steel temperature increases gradually and the signal from both measuring points is almost identical. At the same time, the tundish beingfedthrough a modified ladle shroud supports the asymmetry of the hydrodynamic system, presenting itself as the time shift in the signal (increasing the temperature in the molten steel volume) from measuring point 2. An interesting phenomenon is the balancing of this difference in the system as a consequence of the recirculation area, growing along with the temperature gradient, located at the bottom of the tundish feeding area from outlet No. 1 (Figure 4b,c). The preference for the molten steel flow towards outlet No. 1 is reduced as a consequence of the enlarged recirculation area (Figure 3h). Figure 4d presents the thermal validation of the numerical and physical models for a temperature gradient of 35 K. The water temperature change in the physical model was recorded at measuring point No. 1. The results were highly consistent for both ladle shroud options. 

**Figure 5:**
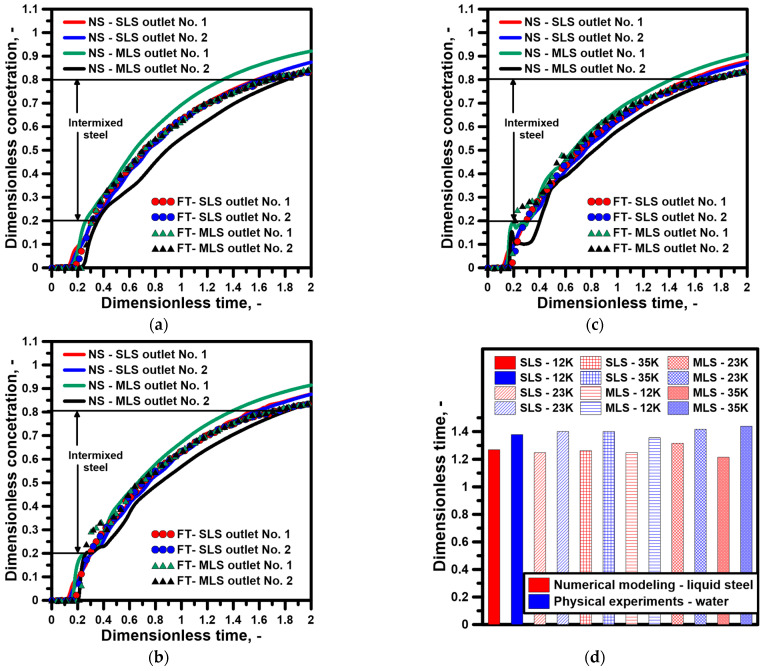
Intermixing process evolution for both ladle shrouds: (**a**) liquid steel ΔT = 12 K, (**b**) liquid steel ΔT = 23 K, (**c**) liquid steel ΔT = 35 K, (**d**) average dimensionless time for intermixing criterion.

Figure 5 presents the results of the marker concentration distribution, describing the process of the subsequent casting of two grades of steel with different chemical compositions. The F curve further supports the reduction in the difference within the hydrodynamic system between the two outlets as a consequence of the evolution of the recirculation area on outlet No. 1’s side and its decay on outlet No. 2’s side (Figure 5b,c). The curves obtained in the course of the experiments on the water model for both outlets show significantly smaller differences than those registered in computer simulations for a tundish with a modified ladle shroud. For the standard ladle shroud, the momentum of the feed stream is the main source of influence on the hydrodynamic structure in the tundish; hence, the results obtained with the two techniques are more consistent. For the purpose of a quantitative assessment, the time of the intermixing steel was calculated, confirming that in the case of a standard ladle shroud, the change in temperature gradient within the measured range does not significantly affect the intermix range. In the case of an MLS, the higher the temperature gradient, the longer the time for the intermixed steel (Figure 5d). However, for a tundish with an MLS and a temperature gradient of 35 K, this tendency is not supported by the numerical model. In the case of the numerical model, during the time period when the F concentration distribution is uniquely flattened, the F concentration obtained during the computer simulations is characterized by values nearly 50% lower, which leads to the shrinking of the amount of intermixed steel. 

### 4.2. Numerical Simulation—Influence of Heat Exchange between Liquid Steel and Environment

**Figure 6:**
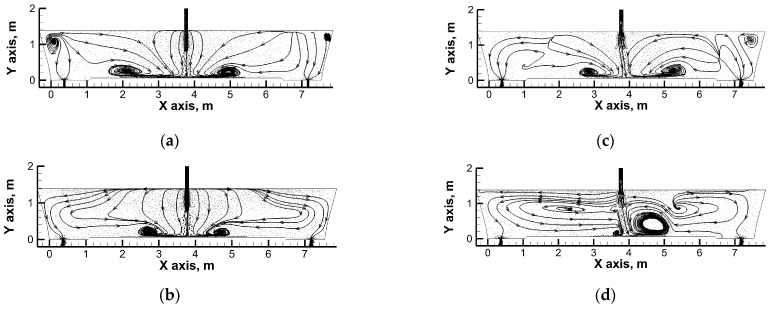
Liquid steel behavior: (**a**) steady-state conditions in the tundish with SLS, (**b**) transient conditions at 0.54 Dt and ΔT = 35 K in the tundish with SLS, (**c**) steady-state conditions in the tundish with MLS, (**d**) transient conditions at 0.54 Dt and ΔT = 35 K in the tundish with MLS.

**Figure 7:**
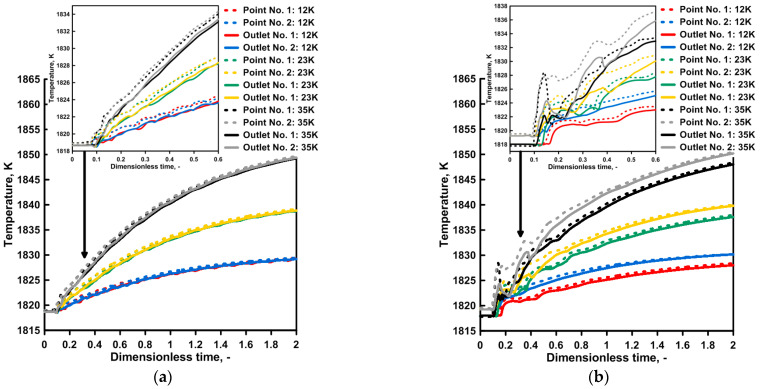
Temperature evolution during casting: (**a**) tundish with SLS, (**b**) tundish with MLS.

In the second phase of the study, the exchange of heat between molten steel, the tundish, and the environment was taken into account. The capacity of the tundish was changed as well, and the tundish was filled with molten steel weighing 75 Mg, with the same distance from the ladle shroud to the bottom of the tundish. The average theoretical residence time for the higher capacity was 1104 s. Figure 6 presents the distribution of the molten steel paths in a tundish with a standard and modified ladle shroud, for a non-dimensional time of 0.54 and temperature gradient of 35 K. When a standard ladle shroud is used, additional recirculation areas are observed in the higher capacity tundish within the outlet’s area (Figure 6a). For the same tundish and the largest temperature gradient considered, the molten steel stream distribution on the considered plane is similar to that of the 60 Mg tundish (Figure 6b). In a tundish fed through a modified ladle shroud, the feed stream turns toward outlet No. 2 (Figure 6c). This turn leads to an evolution of the recirculation area which is the largest for a temperature gradient of 35 K (Figure 6d), like in the case of the 60 Mg tundish. Figure 7 presents the distribution of steel temperature values at measuring points No. 1 and No. 2 and at the outlets of the tundish. If the exchange of heat with the environment is taken into account, the difference in the molten steel temperatures between the feed stream and the outlets is 5 K. When the process of filling the tundish with a hotter steel batch is initiated in a tundish with a standard ladle shroud, the steel temperature increases gradually at the tundish’s outlets. After a time of 2 Dt, the temperature rise is 11, 21 and 31 K for the temperature gradients under consideration. These values are 4 K lower on average than in the case of a simulation not taking into account the exchange of heat with the environment, due to the fire-resistant lining of the tundish and the tundish powder. In a tundish with an MLS, the temperature growth tendency at the outlets is similar, save that it is more dynamic during the initial phase of molten steel mixing. The level of reheating of molten steel in a tundish with a modified ladle shroud is similar to that of a tundish with an SLS. However, in a tundish with a modified ladle shroud, there is a 2 K difference in the molten steel temperature between the outlets of the tundish, so that the steel flowing out through outlet No. 2 is hotter. The molten steel temperature difference registered at the measuring points and at the particular outlets is practically the same, which supports the paths of the streams running to the outlets, which are flowing in parallel to the side walls of the tundish.

**Figure 8:**
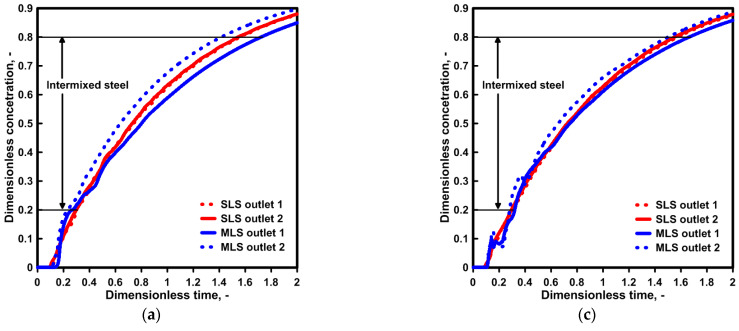

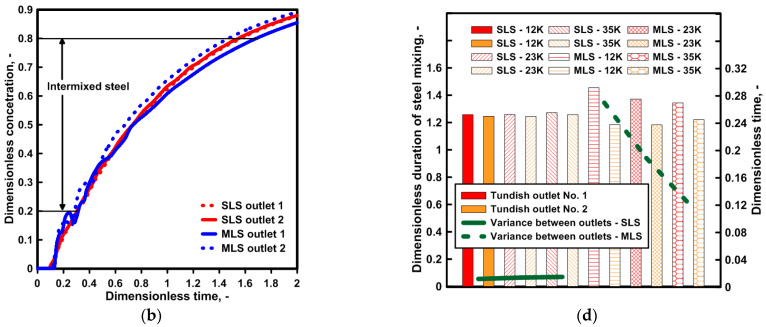
Intermixing criterion for both ladle shrouds: (**a**) liquid steel ΔT = 12 K, (**b**) liquid steel ΔT = 23 K, (**c**) liquid steel ΔT = 35 K, (**d**) time of mixing two grades of steel.

Figure 8 presents the type F residence time distribution and the computed span of the time of the intermixing steel. When an SLS is used, there are no significant differences in the mixing level of the two grades of steel. The difference in the time is 14 s on average. When an SLS is used, the level of steel reheating does not affect the process of mixing two grades of steel with a different chemical composition. In a tundish fed via an MLS, the progressing evolution of the steel recirculation area on the side of outlet No. 2, as a result of the occurrence of a temperature gradient, intensifies the asymmetry of the steel stream, initiated by a turn of the feed stream. For a temperature gradient of 35 K, the increasing asymmetry of the stream structure reduces the difference in the time of the intermixing steel between outlets No. 1 and No. 2 by over 50%, from 298 s to 135 s. 

### 4.3. Numerical Simulations—Influence of Initial Liquid Steel Temperature 

**Figure 9:**
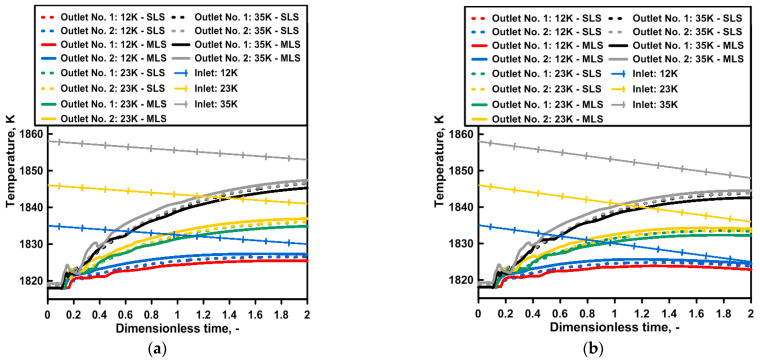
Temperature evolution during casting (phenomenon of liquid steel cooling in the ladle): (**a**) tundish with SLS, (**b**) tundish with MLS.

**Figure 10:**
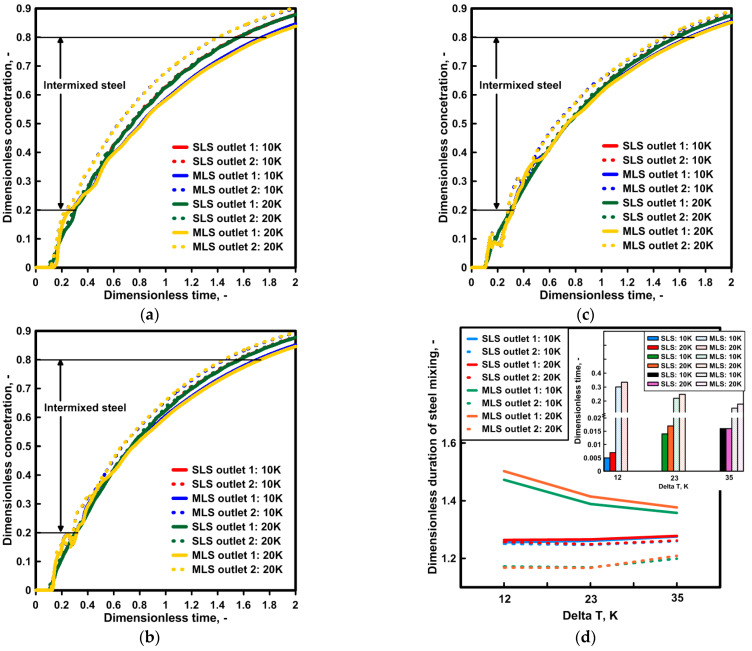
Intermixing criterion for both ladle shrouds with linear decrease initial liquid steel temperature by 10 K and 20 K: (**a**) liquid steel ΔT = 12 K, (**b**) liquid steel ΔT = 23 K, (**c**) liquid steel ΔT = 35 K, (**d**) time of mixing two grades of steel.

In the third phase of our study, the molten steel feeding the tundish was gradually cooling in the ladle, so that the feed stream entering the tundish was decreasing in value as a function of passing time (Figure 9).After the lapse of non-dimensional time Dt = 2, the molten steel entering the tundish was 6 K and 11 K cooler for the considered degrees of molten steel cooling in the ladle. A more intensive cooling process degrades the process of molten steel heating in the tundish by 2 K on average. Hence, for temperature gradients of 12 K, 23 K, and 35 K on the outlets of a tundish, the steel reaching the continuous casting molds increased by 6 K, 15 K, and 26 K, respectively. For a tundish with a modified ladle shroud, adifference of 2 K between the outlets is maintained.Based on the results obtained, type F residence time distribution curves were developed for the hydrodynamic system under consideration (Figure 10). The qualitative assessment demonstrated no significant differences in the distribution of marker concentration as a function of time depending on the molten steel cooling rate in the ladle. Hence, the basis for the assessment of the impact of thermal conditions on the hydrodynamic system and its susceptibility to the evolution of the hydrodynamic structure was the span of time of mixing two steel grades for the particular outlets of the tundish. The temperature gradient has no effect on the hydrodynamic structure when the tundish is fed through a standard ladle shroud. Also, if a modified ladle shroud is used in casting at the highest level of the reheating of molten steel, the development of a recirculation area at the bottom of the tundish leads to a reduction in the difference in the steel mixing time between the outlets of the tundish. The initiation of the molten steel cooling process in the ladle lowers the degree of molten steel reheating, reduces the temperature gradient in the tundish workspace and therefore reduces the effect of natural convection forces on the evolution of molten steel recirculation areas. Hence, even with 12 K of reheating, the difference in time between both tundish outlets for a cooling degree of 20 K was 370 s. For a temperature gradient of 35 K, for this degree of molten steel cooling in the ladle, the difference in time between the outlets decreased to 196 s. However, this value is 1 min higher than that in the case of no molten steel cooling in the ladle. 

## 5. Results Discussion

**Figure 11:**
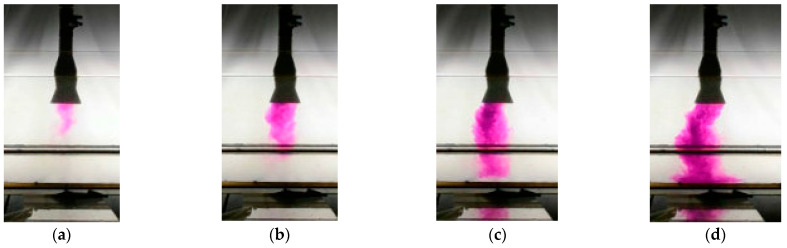
Asymmetric behavior of water stream during pouring of tundish model for water ΔT = 5 K: (**a**) 0.002 Dt after the ladle gate opens, (**b**) 0.005 Dt after the ladle gate opens, (**c**) 0.007 Dt after the ladle gate opens, (**d**) 0.01 Dt after the ladle gate opens.

The numerical model used in our computer simulations, based on the averaged Navier–Stokes equation and realizable k-epsilon model of turbulence, does not offer a full representation of the non-steadiness of the feed flux, particularly in the case of ladle shrouds which have the function of slowing down the momentum of the feed stream. Figure 11 shows the evolution of the feed stream during the experiments on a water model with atemperature delta at 5 K. The feed stream tends to turn towards outlet No. 1 and towards outlet No. 2 in alternation to finally reach a relatively symmetrical distribution at the bottom toward the shroud axis. In the case of numerical simulations, the model demonstrated a strong preference of the stream to turn towards outlet No. 1 or outlet No. 2. The emergence of a prominent asymmetry in the tundish initiates the development of separate hydrodynamic structures in the working volume of the tundish. The evolution of these areas is not only determined by the momentum of the feed stream but also by natural convection. In a tundish with a standard ladle shroud and a fully isolated system in terms of the heat exchange with the environment, the average flow rate of molten steel in the tundish was 0.0393 m/s. With a growing level of molten steel reheating, this value increases to 0.0427 m/s and 0.0429 m/s for temperature gradients of 23 K and 35 K, respectively. The use of a modified ladle shroud in the tundish reduces the average molten steel flow rate in the tundish to anaverage of 0.0239 m/s. Based on Equation (4), the value of Bu was determined, which, if above 5, indicates that natural convection forces have a strong influence on the hydrodynamic system.

(8) $$Bu=\frac{\beta gL\Delta T}{u_{ave}^{2}}$$

where: *u_ave_* is the average liquid steel velocity, *g* is the gravitational force, *L* is the steel level in the tundish, and Δ*T* is the difference in temperature between the inlet and outlet.

For a tundish with a standard ladle shroud, the value of the Bu number was 8.68, whereas for a system in a tundish with a modified ladle shroud, it was 23.49 already at a reheating level of just 12 K. Hence, for both shroud options, the system is strongly susceptible to modification by natural convection forces. This susceptibility is nearly three times stronger if a modified ladle shroud is used, being the source of the non-steady behavior of the feed stream. Hence, the local disturbances (temperature gradients, flow directions) occurring in the hydrodynamic system are the reason enhance the asymmetry flow in the working volume of the tundish. 

## 6. Summary

Based on laboratory investigations, including computer simulations and water model experiments, the effect of the level of the reheating of the molten steel on the evolution of the hydrodynamic system was verified. From our obtained results, the following was found:

▪ The use of the proposed modified ladle shroud during casting via a two-strand slab tundish decreases the average velocity of the liquid steel flow by over 40%, changing the steel flow paths more and more in the horizontal direction.
▪ The asymmetrical impact of the steel feeding stream at the bottom of the tundish favored the phenomenon of steel recirculation. Moreover, the higher the degree of molten steel reheating in the ladle furnace, the stronger the evolution of the recirculation structures in the tundish during the continuous steel casting process when the tundish is pouring via the proposed modified ladle shroud.
▪ The residence time distribution curve type of F is useful to record the hydrodynamic structure’s evolution during the continuous casting process. From the obtained results, the maximum average time of mixing the steel is shorter by 77 s for the tundish pouring via a standard ladle shroud than that when an MLS is used. Moreover, the standard ladle shroud gives more stable conditions for all the considered thermal conditions for the liquid steel feeding stream.
▪ Increasing the temperature of liquid steel in the ladle decreases the divergence in the mixing time between the outlets in the tundish with an MLS by over 50%. This tendency is lowered by up to 8% when the process of the natural exchange of heat between the molten steel and the lining of the tundish and the ladle, as well as the slag phase and the atmospheric air, is considered.
▪ When a tundish is fed through this type of MLS, the momentum of the stream is slowed down, which creates the conditions for the emergence of an asymmetrical flow within the working tundish volume. This is particularly disadvantageous for multiple-outlet tundishes. Local asymmetry may evolve, thus deepening the issue of the asymmetric flow. In the presented work, the stronger evolution of recirculation structures at one side of the pouring zone reduced the influence of the asymmetric behavior of the feeding stream on the divergence in the mixing process—the system strives to achieve a state of equilibrium. Because the tundish works under turbulent conditions, it is obvious control hydrodynamic conditions should be used. Therefore, future investigations into the stability of hydrodynamic conditions using advanced flow control devices in a two-strand tundish with this type of modified ladle shroud are needed.

## Figures and Tables

**Table 1:** Considered variants of continuous casting process.

Case No.	Type of Ladle Shrouds	Heat Exchange between Tundish and Environment	Liquid Steel Delta T, K	Cooling Level of Liquid Steel in the Ladle, K
1	SLS	not considered	12	0
2	SLS	not considered	23	0
3	SLS	not considered	35	0
4	MLS	not considered	12	0
5	MLS	not considered	23	0
6	MLS	not considered	35	0
7	SLS	considered	12	0
8	SLS	considered	23	0
9	SLS	considered	35	0
10	MLS	considered	12	0
11	MLS	considered	23	0
12	MLS	considered	35	0
13	SLS	considered	12	10
14	SLS	considered	12	20
15	SLS	considered	23	10
16	SLS	considered	23	20
17	SLS	considered	35	10
18	SLS	considered	35	20
19	MLS	considered	12	10
20	MLS	considered	12	20
21	MLS	considered	23	10
22	MLS	considered	23	20
23	MLS	considered	35	10
24	MLS	considered	35	20

## Data Availability

Data Availability after contact with corresponding Author.
